# Adsorption of Paraquat by Poly(Vinyl Alcohol)-Cyclodextrin Nanosponges

**DOI:** 10.3390/polym13234110

**Published:** 2021-11-25

**Authors:** Ekkachai Martwong, Santi Chuetor, Jatupol Junthip

**Affiliations:** 1 Division of Science (Chemistry), Faculty of Science and Technology, Rajamangala University of Technology Suvarnabhumi, Phra Nakhon Si Ayutthaya 13000, Thailand; ekkachai.m@rmutsb.ac.th; 2 Department of Chemical Engineering, Faculty of Engineering, King Mongkut’s University of Technology North Bangkok, Bangkok 10800, Thailand; santi.c@eng.kmutnb.ac.th; 3 Faculty of Science and Technology, Nakhon Ratchasima Rajabhat University, Nakhon Ratchasima 30000, Thailand

**Keywords:** paraquat, adsorption, nanosponges, cyclodextrin, citric acid, poly(vinyl alcohol), wastewater pollution, pseudo second-order, Langmuir isotherm, crosslinked polymer

## Abstract

The contamination of hydrosoluble pesticides in water could generate a serious problem for biotic and abiotic components. The removal of a hazardous agrochemical (paraquat) from water was achieved by adsorption processes using poly(vinyl alcohol)-cyclodextrin nanosponges, which were prepared with various formulations via the crosslinking between citric acid and β-cyclodextrin in the presence of poly(vinyl alcohol). The physicochemical properties of nanosponges were also characterized by different techniques, such as gravimetry, thermogravimetry, microscopy (SEM and Stereo), spectroscopy (UV-visible, NMR, ATR-FTIR, and Raman), acid-base titration, BET surface area analysis, X-ray diffraction, and ion exchange capacity. The C10D-P2 nanosponges displayed 60.2% yield, 3.14 mmol/g COOH groups, 0.335 mmol/g β-CD content, 96.4% swelling, 94.5% paraquat removal, 0.1766 m^2^ g^−1^ specific surface area, and 5.2 × 10^−^^4^ cm^3^ g^−^^1^ pore volume. The presence of particular peaks referring to specific functional groups on spectroscopic spectra confirmed the successful polycondensation on the reticulated nanosponges. The pseudo second-order model (with R^2^ = 0.9998) and Langmuir isotherm (with R^2^ = 0.9979) was suitable for kinetics and isotherm using 180 min of contact time and a pH of 6.5. The maximum adsorption capacity was calculated at 112.2 mg/g. Finally, the recyclability of these nanosponges was 90.3% of paraquat removal after five regeneration times.

## 1. Introduction

The contamination of pesticides in water is a significant environmental topic that threatens both the ecosystem and public health. Paraquat (PQ) or 1,1′-Dimethyl-4,4′-bipyridinium dichloride) is a non-selective herbicide for plantation or defoliation that is a very hydrosoluble agrochemical [1,2]. The appearance of this dangerous herbicide causes different negative effects on the environment [3,4] and heath [5,6,7,8,9,10,11]. Physical [12,13], biological [14,15], and chemical treatments [16,17,18,19,20,21,22] have all been reported in the literature for PQ removal. An adsorption process has recently been investigated for PQ removal, which was applied for both organic and inorganic materials such as bio-based material [23,24,25], bentonite [26,27], microorganisms [28], activated carbon [29,30], kaolin [31], pillararene [32,33], calixarene [34,35,36], graphene oxide [37], carbon nanotubes [38,39], silica [40,41], magnetic adsorbent [42,43], montmorillonite [44,45], cellulose nanofiber [46,47], and cyclodextrin [48].

Adsorbents based-cyclodextrin have been widely used for environmental applications [48,49,50,51,52,53,54,55] according to the special properties of cyclodextrin. β-cyclodextrin (β-CD) is a cyclic oligosaccharide containing seven units of d-glucose connected by α-(1,4) glycosidic linkages, which shows a well-defined structure with a hydrophilic exterior and a hydrophobic cavity. The extraordinary property of this supramolecular molecule leads to the entrapment of organic compounds with a suitable size into the cyclodextrin cavity by the formation of an inclusion complex according to the reversible reaction via host-guest interaction. Moreover, the development of cyclodextrin polymer has widely been established by various types of polymerization so as to improve the solubility for both the cyclodextrin polymer and the guest molecule and enhance the adsorption capacity towards organic compounds. Insoluble cyclodextrin polymers and their derivatives have been innovated for environmental applications [48,49,50,51,52,53,54,55,56,57,58,59,60,61,62,63,64,65,66,67,68,69,70,71] and other fields [72,73,74,75,76]; they are also called “cyclodextrin nanosponges” because these nanomaterials display a sponge-like structure and a hyperbranched network with three-dimensional form and have a specific role in forming a functional crosslinker or other chemical reactants [52]

Citric acid (CTR) was used as a green trifunctional crosslinker to create the bridged framework, which represented, simultaneously, an anionic character by the presence of non-crosslinked carboxylic groups. This type of cyclodextrin nanosponge has been enormously studied for environmental remediation [48,59,63,64,65,66,67,68,69,70]. Poly(vinyl alcohol) is a hydrosoluble, semi-crystalline and synthetic polymer with an excellent degree of swelling, nontoxicity, biodegradability, and suitable mechanical properties that can also react with citric acid or other crosslinking agents to create interesting water-insoluble three-dimensional polymers. Therefore, the appearance of poly(vinyl alcohol) in cyclodextrin nanosponges could promote the adsorption capacity towards cationic, anionic, and neutral molecules according to the abundance of available hydroxyl groups on the polymeric chains, which displays as the supplementary adsorption sites with these molecules via hydrogen bonding [77].

Cyclodextrin nanosponges crosslinked with citric acid in the presence of poly(vinyl alcohol) was investigated in a previous study so as to elaborate an efficient adsorbent of cyclodextrin nanosponges that were applied to the adsorption of phenol and methylene blue [78] and aniline extraction [79] and the removal of naphthenic acids [80]. Nevertheless, this type of nanosponge has never been declared for PQ adsorption. In this research, poly(vinyl alcohol)-cyclodextrin nanosponges were first prepared by reticulation of CTR with β-CD and/or PVOH, and their physicochemical attributes were characterized by different techniques. The kinectics, isotherm, and the reusability of PQ adsorption were then investigated.

## 2. Materials and Methods

### 2.1. Materials

Citric acid monohydrate (RCI labscan, Bangkok, Thailand), β-cyclodextrin (Acros Organics, Geel, Belgium), sodium hypophosphite (Acros Organics, Geel, Belgium), Poly(vinyl alcohol) M_w_ = 89,000–98,000 with 99+% hydrolyzed (Sigma-Aldrich, Saint Louis, MO, USA), and paraquat dichloride hydrate (Sigma-Aldrich, Saint Louis, MO, USA) were acquired from commercial sources. Other chemicals used in this work were analytical grade. Ultrapure water was employed for all experiments.

### 2.2. Nanosponges Preparation

The nanosponges were synthesized by the previous method [48]. The mixture containing the different compositions of β-CD, CTR, and PVOH, as described in Table 1, and 3% *w*/*v* sodium hypophosphite used as catalyst was dissolved in 100 mL of water and heated under magnetic agitation before transferring to a 500 mL round-bottom flask. This was then placed into a rotary evaporator (Heidolph Hei-VAP Advantage, Schwabach, Germany) at 70 °C to entirely remove water under vacuum until a solid mixture was obtained, after which it was crosslinked in a rotary evaporator at 180 °C for 30 min under vacuum. The nanosponges were washed with water and ethanol before drying at 120 °C to eliminate all of the solvents. Finally, the nanosponges were crushed with a mortar and a pestle to obtain a fine powder.

### 2.3. Nanosponges Characterization

The nanosponges were characterized by various physicochemical techniques. The percentage yield was determined by the ratio between the mass of the final product and the mass of the initial reactants. The scanning electron microscopy (SEM) investigation was manipulated on a JEOL 6010 electron microscope (Tokyo, Japan) with an acceleration voltage of 15 kV. The morphology of the sample was observed by a Nikon SMZ745T stereomicroscope (Melville, NY, USA) equipped with a DS-Fi3 digital camera. The thermogravimetric analysis (TGA) experiments were run in an alumina pan with a Thermal Analyzer—STA 449 F3 (NETZSCH, Waldkraiburg, Germany) from ambient to 500 °C with a heating rate of 10 °C min^−^^1^ under nitrogen. Fourier transform infrared spectroscopy (FTIR) experiments were operated on a Bruker Tensor 27 FTIR (Billerica, MA, USA), which accumulated from 64 scans in the 700–4000 cm^−^^1^ range with a resolution of 4 cm^−^^1^, using attenuated total reflection (ATR) mode. Raman spectroscopy experiments were manipulated on a Cora 5700 Raman spectrometer (Bangkok, Thailand) using 300 mW of laser power, 785 nm of laser wavelength, 10 s of integration time, and 100–2000 cm^−1^ of spectral range, with a resolution of 9 cm^−1^. The nitrogen adsorption–desorption isotherm was conducted at 77 K using a BELSORP-mini II analyzer (Bel Japan, Inc., Haradanaka Toyonaka, Japan) and 150 mg of nanosponges, and it was then degassed under vacuum at 140 °C for 6 h. The surface area was acquired by the BET method and the pore volume and the average pore diameter were quantified by the Barret–Joyner–Halenda (BJH) model. ^13^C NMR (nuclear magnetic resonance) spectra were registered on a Bruker Ascend 400 WB spectrometer (Massachusetts, USA) at 100.62 MHz and 298 K using the magic angle spinning (MAS) technique, glycine as a reference, a delay time of 8 s, and a contact time of 1.5 ms. X-ray Diffraction (XRD) spectra were performed on a Rigaku SmartLab SE X-ray diffractometer (Tokyo, Japan) using an angular range (2θ) between 10° to 80°, 10°/min of scan speed, 0.02° of step width of 0.02° 40 kV of generator voltage, and 50 mA of generator current.

The determination of β-CD was executed by photometric titration using phenolphthalein. The calibration curve was firstly established by taking 0, 2, 4, 6, 8, and 10 mg of β-CD into each volumetric flask (25 mL) before the addition 1 mL of phenolphthalein (0.68 mmol L^−1^), 2.5 mL of Na_2_CO_3_ (1 mol L^−1^), and 21.5 mL of water. After agitation (150 rpm) for 24 h, the aliquot was measured at 552 nm by UV-Vis spectrophotometer (GENESYS 10S, Thermo Scientific, Vantaa, Finland). The measurement of the β-CD cavities of the nanosponges was performed in the same process as mentioned above using 20 mg of nanosponges instead of β-CD. The β-CD content was expressed in mmol per gram of nanosponges.

The quantification of the ion exchange capacity (*IEC*) of nanosponges was executed by pH-metric titration. The nanosponges (0.1 g) were immersed into 50 mL of 2% *w*/*v* calcium acetate solution for 4 h under stirring at 150 rpm. After withdrawing the sample, the solution containing acetic acid was titrated by NaOH solution (0.05 M) using phenolphthalein as an indicator. The *IEC* was expressed in mmol of COOH groups per gram of nanosponges using the following equation:

(1) $$IEC\;\left(mmol/g\right)=\frac{C_{\mathrm{NaOH}\;}\times {\;V}_{\mathrm{NaOH}}}{m}$$

where V_NaOH_ and C_NaOH_ correspond, respectively, to the equivalent volume (mL) and concentration (mol/L) of NaOH. The symbol m refers to nanosponges weight (g). Experiments were operated in triplicate.

The swelling behavior of the nanosponges was investigated by solution uptake determination. Ultrapure water (10 mL) was added to a test tube containing 100 mg of nanosponges at 30 °C under agitation of 150 rpm. After 24 h of immersion, the swollen sample was removed and drained before weighing. The swelling was calculated in percent using the following equation:

(2) $$Swelling\;\left(\%\right)=\frac{W_{2}-W_{1}}{W_{1}}\times 100$$

where W_1_ and W_2_ refer, respectively, to dried and swollen nanosponges. Experiments were executed in triplicate.

### 2.4. Adsorption Study

#### 2.4.1. Preliminary Adsorption Study

An amount of 10 mL of PQ solution with a 25 mg/L of initial concentration at different pH (2, 3, 4, 5, 6.5, 8, 9, and 10), which was adjusted with 0.1 M HCl and 0.1 M NaOH, was added to a test tube containing 20 mg of nanosponges under agitation (150 rpm) for 180 min at 30 °C. The amount of PQ was quantified by UV-Vis spectrophotometer (GENESYS 10S, Thermo Scientific, Vantaa, Finland) at 257 nm. The paraquat removal was expressed in percentage using the following equation:

(3) $$\%\;\mathrm{Paraquat}\;\mathrm{removal}\;=\frac{{(C}_{0}-C_{t})}{C_{0}}\times 100$$

where C_0_ and C_t_ relate, respectively, to the initial and real-time concentration of PQ. Experiments were performed in triplicate. The adsorption capacity (Q) was also exhibited using the following equation:

(4) $$\mathrm{Adsorption}\;\mathrm{capacity}\;\left(\mathrm{mg}/g\right)=\frac{{(C}_{0}-C_{t})\;\times \;V}{m}$$

where C_0_ and C_t_ relate, respectively, to the initial and real-time concentration of PQ, V refers to solution volume, and m stand for nanosponges mass.

The different types of nanosponge were also tested to evaluate the adsorption efficiency. An amount of 10 mL of PQ solution with a 25 mg/L of initial concentration and optimal pH was poured into a test tube containing 20 mg of nanosponges under agitation (150 rpm) for 180 min at 30 °C. The quantity of PQ was measured as explained previously.

#### 2.4.2. Kinetics Study

An amount of 10 mL of PQ solution with a 25 mg/L initial concentration and optimal pH was poured into a test tube containing 20 mg of nanosponges under agitation of 150 rpm at different times (15, 30, 45, 60, 120, 180, and 300 min) at 30 °C. The measurement of PQ has been described in the previous section. Experimental data were then fitted with two kinetics models:

Pseudo first-order model:

ln (Q_e_ − Q_t_) = ln Q_e_ − k_1_t (5)

Pseudo second-order model:

(6) $$\frac{t}{Q_{t}}=\frac{1}{k_{2}Q_{e}^{2}}+\frac{1}{Q_{e}}t$$

where Q_e_ and Q_t_ are the quantity of PQ adsorbed (in mg/g) at equilibrium and at time t, respectively, k_1_ (/min) and k_2_ (g/mg·min) are adsorption rate constant, and t is contact time (min). Experiments were performed in triplicate.

The quantity of paraquat adsorbed versus the square root of time was plotted using the intraparticle diffusion model as the following equation:

Intraparticle diffusion model:

Q_t_ = k_3i_t^0.5^ (7)

where Q_t_ are the quantity of PQ adsorbed (in mg/g) at time t, respectively, k_3i_ (g/mg·min^0.5^) are adsorption rate constant, and t is contact time (min). Experiments were performed in triplicate.

#### 2.4.3. Isotherm Study

An amount of 10 mL of PQ solution with different initial concentration (25, 50, 150, 250, and 300 mg/L) and optimal pH was poured into a test tube containing 20 mg of nanosponges under agitation of 150 rpm at equilibrium and 30 °C. The quantification of PQ has been previously mentioned. Experimental data were then fitted with two isotherm models:

Langmuir isotherm:

(8) $$\frac{C_{e}}{Q_{e}}=\frac{1}{K_{L}Q_{m}}+\frac{C_{e}}{Q_{m}}$$

Freundlich isotherm:

(9) $$\ln\;\mathrm{Qe}\;={\;\ln\;K}_{F}+\frac{1}{n}\ln\;\mathrm{Ce}$$

where C_e_ is the equilibrium concentration of PQ, Q_e_ is the amount of PQ adsorbed (in mg/g) at equilibrium, Q_m_ is the theoretical maximum adsorption capacity (in mg/g), K_L_ is the Langmuir isotherm constant, K_F_ is the Freundlich isotherm constant, and 1/n is heterogeneity factor.

The chi-square test was also used as a statistical analysis so as to access the suitability of isotherm equations to the experimental data. The chi-square value (χ^2^) was expressed by the following equation:

Chi-square value:

(10) $$\chi 2=\sum \frac{{{(Q}_{e,\exp}{-\;Q}_{e,\mathrm{cal}})}^{2}}{Q_{e,\mathrm{cal}}\;}$$

where Q_e,exp_ is the amount of PQ adsorbed (in mg/g) at equilibrium calculated from the experimental data and Q_e,cal_ is the amount of PQ adsorbed (in mg/g) at equilibrium estimated from the models.

#### 2.4.4. Reusability Study

An amount of 10 mL of PQ solution with 25 mg/L of initial concentration and optimal pH was poured into a test tube containing 20 mg of nanosponges under agitation of 150 rpm at equilibrium and 30 °C. The measurement of PQ has been described in the previous section. However, the adsorbent was then separated and regenerated by cleaning in methanol for PQ desorption. After 180 min of soaking, the adsorbent was washed with ultrapure water and reconditioned for sorption in posterior cycles.

## 3. Results and Discussion

### 3.1. Preparation and Characterization of Nanosponges

#### 3.1.1. Physicochemical Properties of Nanosponges

**Figure 1:**
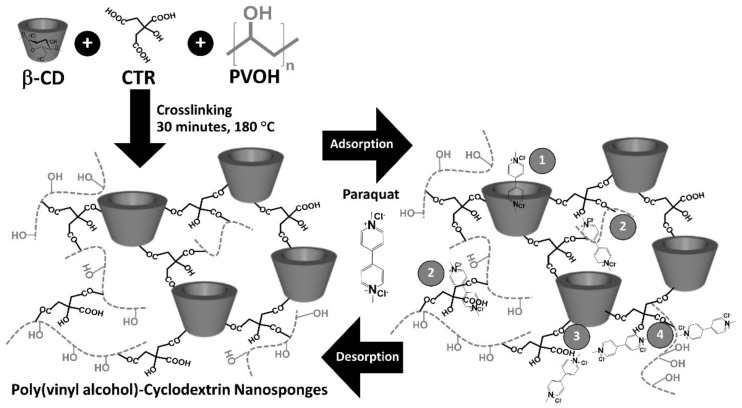
Schematic reaction of poly(vinyl alcohol)-cyclodextrin nanosponges and the possible adsorption mechanism with paraquat.

The poly(vinyl alcohol)-cyclodextrin nanosponges were productively prepared by crosslinking between β-CD and CTR in the presence of PVOH via esterification reaction to create negative charges due to the presence of non-crosslinked carboxylic functions from CTR, which could be dissociated in carboxylate functions, as displayed in Figure 1.

These nanosponges represent the reticulated structure that was established from the polycondensation between various reactants to build the different skeletons such as β-CD-CTR-β-CD, β-CD-CTR-PVOH, and PVOH-CTR-PVOH crosslinked forms. Furthermore, the other conformation also appeared by bridging the available COOH groups from these three skeletons with β-CD and/or PVOH, which again authorized the attachment of PVOH at the border of the crosslinked structure. This segment could be profitable for the adsorption of various molecules by non-covalent interaction on the PVOH section. These anionic nanosponges could remove cationic organic pollutant from wastewater, and paraquat dichloride was chosen for this study to evaluate the removal efficiency of these nanosponges.

As observed in Table 1 for the nanosponges named C2.5D-P2, C5D-P2, and C10D-P2, the reaction yield increased from 52.9% to 60.2% with the quantity of CTR from 2.5% *w*/*v* to 10% *w*/*v*, which also enhanced the ion exchange capacity from 2.13 mmol/g to 3.14 mmol/g, because the presence of higher CTR could enhance the polycondensation between CTR and β-CD. These results are in agreement with the literature report [81]. However, the growth of CTR decreased the swelling from 199.2% to 96.4% due to the strong crosslinking density of new ester bridges in the polymer network, which could result in the structure being more packed, limit the movement of polymer chains, and prevent the penetration of water into the polymer network. This result was in accordance with the results reported in the literature [82,83,84,85]. Moreover, the amount of active β-CD was reduced from 0.365 mmol/g to 0.335 mmol/g with the augmentation of CTR due to the inaccessibility of cyclodextrin cavities, which were sterically hindered by the solidity of the crosslinked structure and also restricted the encapsulation of phenolphthalein into the cyclodextrin cavities. This situation was in agreement with the data reported in the literature [86].

**Figure 2:**
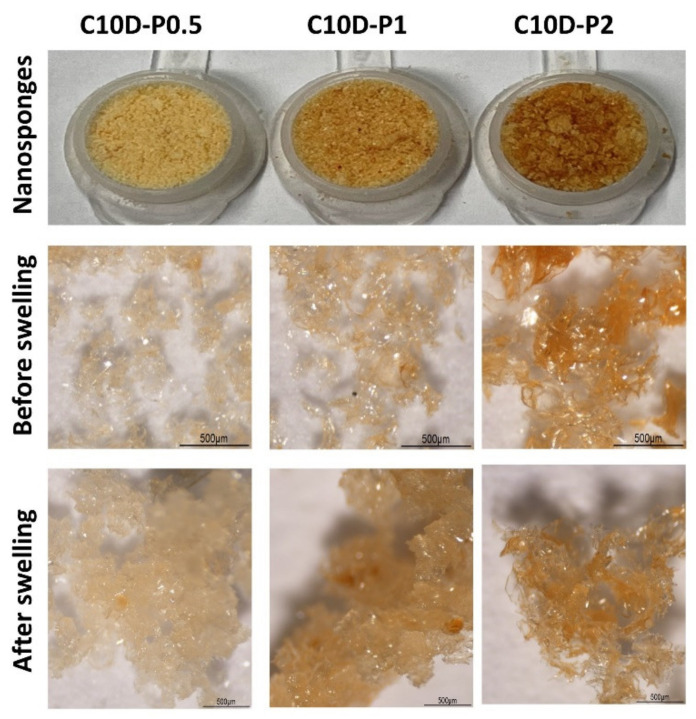
Physical appearance of nanosponges before and after swelling in water.

For the nanosponges named C10D-P0.5, C10D-P1, and C10D-P2, as shown in Table 1, the addition of PVOH could alter the physicochemical properties, which could provoke the reduction of reaction yield (from 68.6% to 60.0%) and the ion exchange capacity (from 3.27 mmol/g to 3.14 mmol/g) because PVOH was increasingly esterified with available COOH functions from CTR so as to form the crosslinked structure, and this event was also competed with the polycondensation between β-CD and CTR. Therefore, the reaction yield was decreased, as reported in the literature [81] and the loss of free COOH functions dropped the ion exchange capacity. Moreover, the addition of PVOH incited the fall of swelling (from 104.1% to 96.4%) according to the reticulated of OH functions from PVOH with obtainable COOH from CTR, which made the nanosponges more compact and prohibited the insertion of water into the polymer system. Surprisingly, the β-CD content was slightly enhanced, from 0.314 mmol/g to 0.335 mmol/g, with the rise of PVOH, which may occurred due to the adsorption of phenolphthalein on the crosslinked PVOH network. The physical appearance of these nanosponges before and after swelling is monitored in Figure 2. A yellow intense coloration was obviously seen with the increased amount of PVOH, which confirms the enhanced crosslinking of PVOH with CTR.

For the systems without cyclodextrin, C10-P0.5, C10-P1, and C10-P2, the summation of PVOH revealed the gain of reaction yield (from 17.3% to 39.9%). Conversely, with the nanosponges containing cyclodextrin, the rise of reaction yield was increased with the quantity of PVOH due to the favorable polymerization between PVOH and CTR for the system in the absence of β-CD, which the quantity of CTR was in excess of and the reaction rate could possibly be improved. The declination of ion exchange capacity (from 4.46 mmol/g to 3.84 mmol/g) and swelling (from 166.7% to 135.3%) was observed due to the addition of PVOH, which enhanced the crosslinking reaction between available COOH groups from the CTR and OH groups from PVOH, cut the anionic characters presented by the COOH groups, and obstructed the infiltration of water into the polymer framework.

#### 3.1.2. SEM and BET Investigation

**Figure 3:**
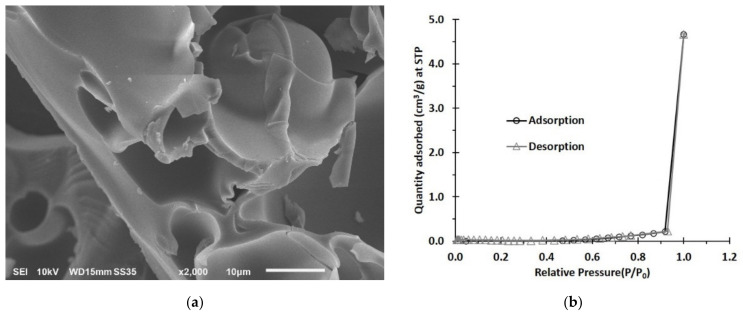
(**a**) SEM image of C10D-P2 nanosponges (**b**) BET isotherm plot of C10D-P2 nanosponges.

The morphology of C10D-P2 nanosponges was monitored at 2000× magnification with 10 μm of full scale; these nanosponges showed a thin layer with a rough surface and very small pores (Figure 3a).

The surface area and pore distribution of nanosponges was determined by BET using a nitrogen adsorption–desorption method (Figure 3b). The C10D-P2 nanosponges exhibited a small specific surface area (0.1766 m^2^ g^−1^), a low pore volume (5.2 × 10^−4^ cm^3^ g^−1^), and a small pore size (4.19 nm), which were all in accordance with that reported in the literature [63]. The presence of PVOH on nanosponges revealed a lower specific surface area and a pore volume because of the CTR-PVOH reticulated structure and the attendance of PVOH at the end of the polymer chains, comparing these values with the citric acid crosslinked cyclodextrin nanosponges, as shown in the literature [48,82].

#### 3.1.3. TGA Analysis

**Figure 4:**
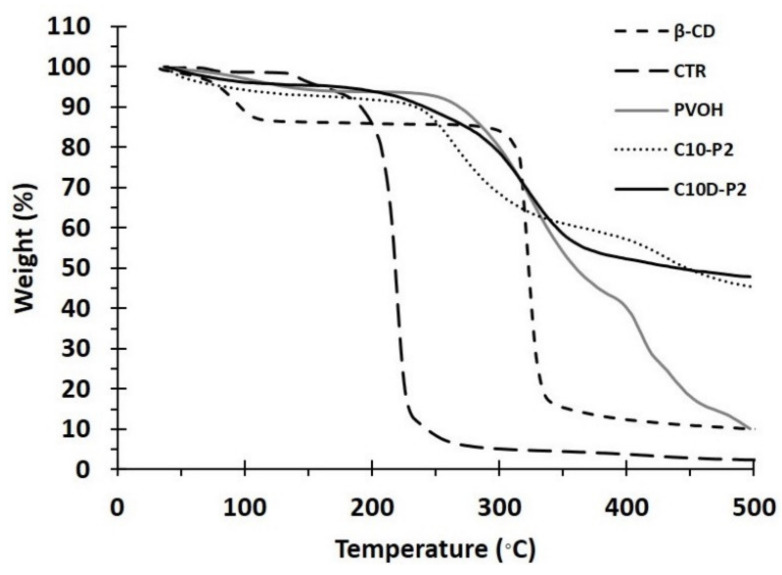
TGA thermograms of nanosponges and reactants.

The thermal study of the nanosponges was evaluated by TGA, as shown in Figure 4, for CTR, PVOH, C10D-P2, C10-P2, and β-CD. The loss of mass below 100 °C represented the material dehydration, which was equal to 1.4%, 2.9%, 3.9%, 5.6%, and 10.7%, respectively. The thermal degradation then started at 136.5 °C, 241 °C, 179 °C, 227 °C, and 296 °C, respectively. The addition of PVOH to the system debased the thermal characteristics for both C10D-P2 and C10-P2 nanosponges. Finally, the gentle decomposition of a residue above 400 °C was thermally stable for C10D-P2 and C10-P2, which displayed the remaining weight at 500 °C of 47.8% and 45.3%, respectively.

#### 3.1.4. ATR-FTIR and Raman Exploration

**Figure 5:**
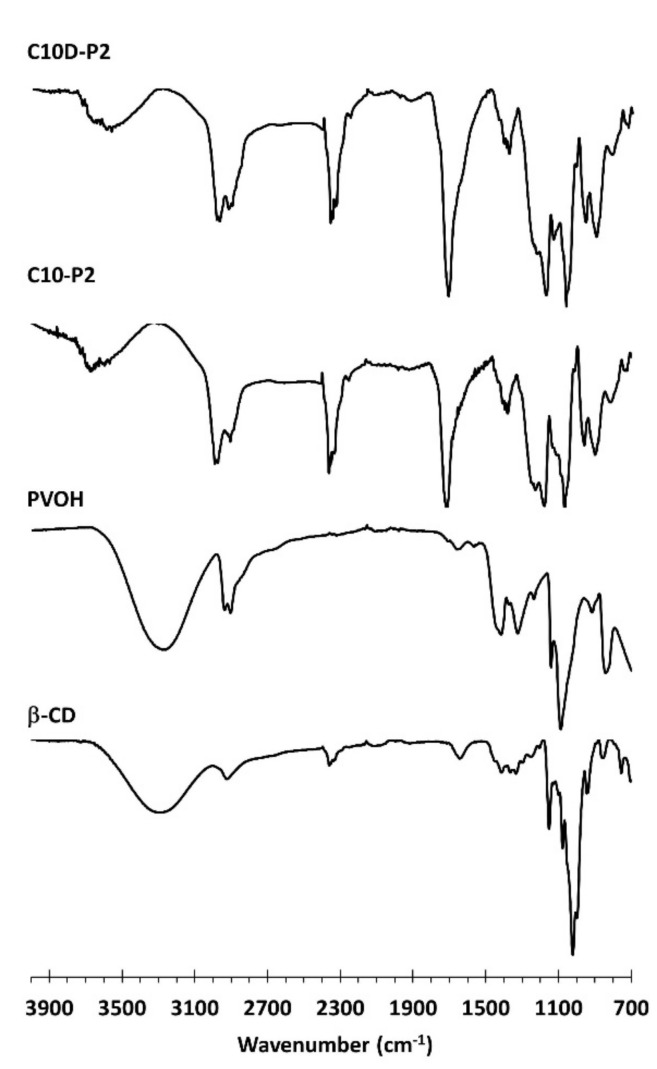
ATR-FTIR spectra of β-CD, C10-P2, and C10D-P2 nanosponges.

ATR-FTIR was conducted to characterize the functional groups presented on nanosponges, as shown in Figure 5. The native β-CD spectra revealed specific peaks at 3288 cm^−1^, attributed to OH stretching, at 2917 cm^−1^, attributed to CH_2_ stretching, at 1152 cm^−1^, attributed to C–C stretching, and at 1021 cm^−1^, attributed to C–O–C stretching of the glycosidic bond, which is in agreement with the data reported in the literature [87]. The PVOH spectra displayed exclusive peaks at 3267 cm^−1^, attributed to OH stretching, at 2939 cm^−1^, attributed to CH stretching, at 2907 cm^−1^, attributed to CH_2_ stretching, at 1417 cm^−1^, attributed to CH bending, at 1325 cm^−1^, attributed to CH wagging, and at 1087 cm^−1^, attributed to C–O–C stretching. There were no obvious peak around 1730 cm^−1^, which confirmed the purity of PVOH with the excellent hydrolysis.

The appearance of a new band was found at 1713 cm^−1^ for both C10D-P2 and C10-P2, which was attributed to C=O stretching of carboxylic and ester functions, which were superposed to each other. This result has also been mentioned in the literature for C10-P2 [88] and C10D-P2 [79]. The extraordinary peak at 1218 cm^−1^, attributed to the C–O–C stretching of ester bonds, has also been noticed and stated in the literature [78]. Therefore, the polycondensation between COOH groups from the CTR and OH groups forming β-CD and PVOH was confirmed by the formation of ester bonds so as to create the three-dimensional framework.

**Figure 6:**
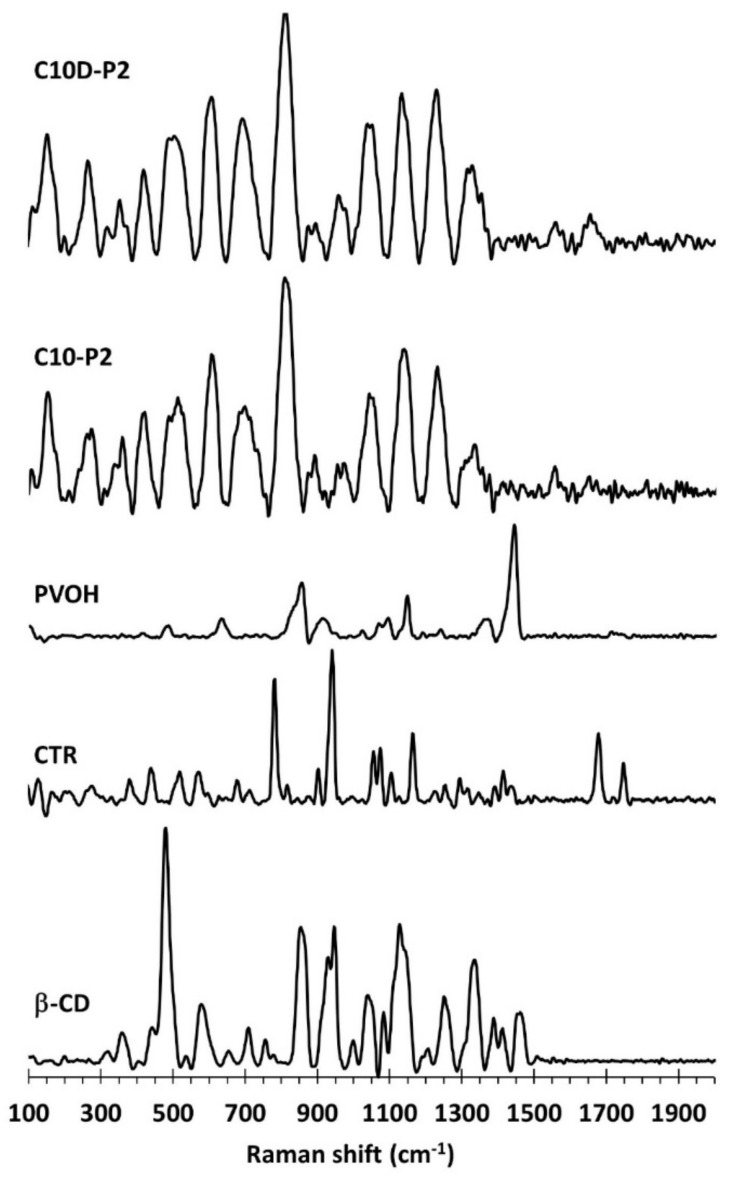
Raman spectra of β-CD, CTR, PVOH, C10-P2, and C10D-P2 nanosponges.

The characterization of the functional groups was also carried out by Raman spectroscopy, as displayed in Figure 6. The pristine β-CD spectra exhibited the characteristic bands at 1463 cm^−1^ for CH deformation, 1414 cm^−1^ for C–O–C symmetric and antisymmetric stretching, 1389 cm^−1^ for CH stretching and wagging, 1338 cm^−1^ for CH_2_ deformation, 1252 cm^−1^ for OH in plane bending and CH_2_ stretching, 1125 cm^−1^ for C–O–C symmetric stretching, 1081 cm^−1^ for C–O–C symmetric and antisymmetric stretching of glycosidic bonds, 1041 cm^−1^ for C–O stretching, 947 cm^−1^ for skeletal mode of α-(1–4) linkage (delocalized mode), 931 cm^−1^ for glucopyranose (C–O–C) skeletal mode of α-anomers, 855 cm^−1^ for OCH side group deformational of d-glucopyranose units, 757 cm^−1^ for d-glucopyranose ring breathing mode, 709 cm^−1^ for CH out of plane bending, 575 cm^−1^ for OH wagging, 479 cm^−1^ for skeletal vibrations of amylose, 439 cm^−1^ for CH stretching, 356 cm^−1^ for OH stretching, 318 cm^−1^ for external C–OH out of plane bending of glucopyranose units, and 155 cm^−1^ for the breathing motions of oxygen atoms in the macrocyclic ring. These results were in agreement with the literature [89,90,91,92].

The citric acid spectra showed the particular peaks at 1750 cm^−1^ for C=O stretching of the central carboxylic groups, 1680 cm^−1^ for C=O stretching of the lateral carboxylic groups, 1468 cm^−1^ for CH_2_ scissors, 1440 cm^−1^ for C–OH deformation, 1347 cm^−1^ for O–CO deformation bending of the carboxylic groups, 1078 cm^−1^ for C–O stretching, 942 cm^−1^ for C–C symmetric stretching, 905 cm^−1^ for C–C bends and OH out-of-plane bending, and 782 cm^−1^ for C_3_CO deformation, as reported in an antecedent study [93,94,95,96].

The PVOH spectra indicated the distinct peaks at 1445 cm^−1^ for CH bending and OH bending, 1376 cm^−1^ for CH bending and OH bending, 1146 cm^−1^ for C–C and C–O stretching, 1097 cm^−1^ for C–O stretching and OH bending, 918 cm^−1^ for C–C stretching, and 857 cm^−1^ for C–C–O stretching, as informed in previous works [97,98,99,100].

The C10D-P2 and C10-P2 nanosponges illustrated similar Raman spectra, which indicated the unique peaks at 1680 cm^−1^ for C=O stretching of the acid and ester groups, 1252 cm^−1^ for CH_2_ stretching, 1146 cm^−1^ for CC and CO stretching, 1060 cm^−1^ for C–O stretching, 966 cm^−1^ for skeletal mode of α-(1–4) linkage (delocalized mode), and 817 cm^−1^ for C–O–C stretching. However, the anticipated peak near 1750 cm^−1^ for C=O stretching of the acid and ester groups was not observed, which might be superposed with the baseline.

#### 3.1.5. XRD and NMR Characterization

**Figure 7:**
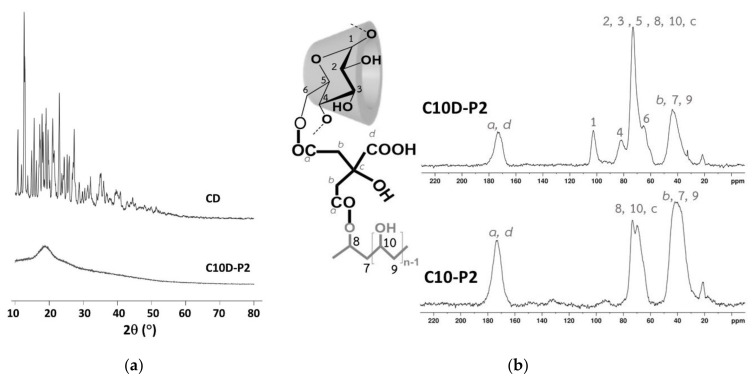
(**a**) XRD spectra of β-CD and C10D-P2 nanosponges; (**b**) ^13^C NMR spectra of C10D-P2 and C10-P2 nanosponges.

The XRD of C10D-P2 and β-CD are shown in Figure 7a. The intense and sharp peaks of β-CD indicate a semi-crystalline structure, where their characteristic peaks can be seen at 12.6°, 17.8°, 19.6°, 22.9°, 24.3°, and 27.2°, and it was indexed to a monoclinic symmetry, as reported in the literature [101]. Nevertheless, no obvious specific peaks were seen in the C10D-P2 nanosponges. The large diffraction peak at 18.3° only appeared due to its amorphous structure according to the polyaddition between CTR and β-CD or CTR and PVOH, which demolished the crystallinity of the β-CD. This amorphous structure could enhance the adsorption capacity regarding pollutants. This result was in agreement with the data reported in the literature, which indicated that the broad band of CTR crosslinked β-CD nanosponges [66].

Both reactants and nanosponges were subjected to ^13^C NMR spectroscopy to show the chemical structure before and after polymerization; the position of various reactants was identically named, as noted in the nanosponges system (Figure 7b). The chemical shifts of different reactants can be described as follows (spectra not shown): β-CD (at 103.0 ppm (for 1), 82.9 ppm (for 4), 78.1 ppm (for 3), 74.9 ppm (for 2 and 5), and 60.7 ppm (for 6)), CTR (at 174.7 ppm (for d), 172.1 ppm (for a), 72.5 ppm (for c), and 42.6 ppm (for b)), and PVOH (at 70.3 ppm (for 9) and 44.5 ppm (for 10)).

The C10D-P2 and C10-P2 nanosponges were characterized by ^13^C NMR spectroscopy so as to verify the polycondensation between CTR and β-CD or CTR and PVOH. The ^13^C spectra of C10-P2 showed characteristic peaks that were marked at 173.0 ppm (for a and d), 72.5 ppm (for 8,10, and c), and 41.8 ppm (for b, 7, and 9), and the polymerization between CTR and PVOH was also confirmed by the change of chemical shift of CTR and PVOH. The C10D-P2 nanosponges exhibited ^13^C specific chemical shifts, which indicated at 173.5 ppm (for a and d), 102.4 ppm (for 1), 81.8 ppm (for 4), 72.9 ppm (for 2, 3, 5, 8, 10, and c), 65.2 ppm (for 6), and 43.8 ppm (for b, 7, and 9). The chemical shift of reactants was changed and the esterification between CTR and β-CD or CTR and PVOH were proven, as reported in the literature [86,102,103].

### 3.2. Adsorption Study

#### 3.2.1. Preliminary Adsorption Study

The influence of pH was initially optimized so as to assess the adsorption performance; the pH of the solutions played an important role in the characteristic properties of both adsorbate and adsorbent. The possible adsorption mechanisms of PQ on the nanosponges was classified into four feasible types, as illustrated in Figure 1: (i) inclusion complex by the encapsulation of PQ into the β-CD cavity via host-guest interaction, (ii) network capture of PQ in the crosslinked structure (both CTR crosslinked PVOH and CTR crosslinked β- CD), (iii) electrostatic interaction between the cationic charge of PQ and the anionic charge of CTR, and (iv) hydrogen bonding between N atoms of quaternary ammonium groups from PQ and H atoms of OH groups from PVOH or CTR [104].

**Figure 8:**
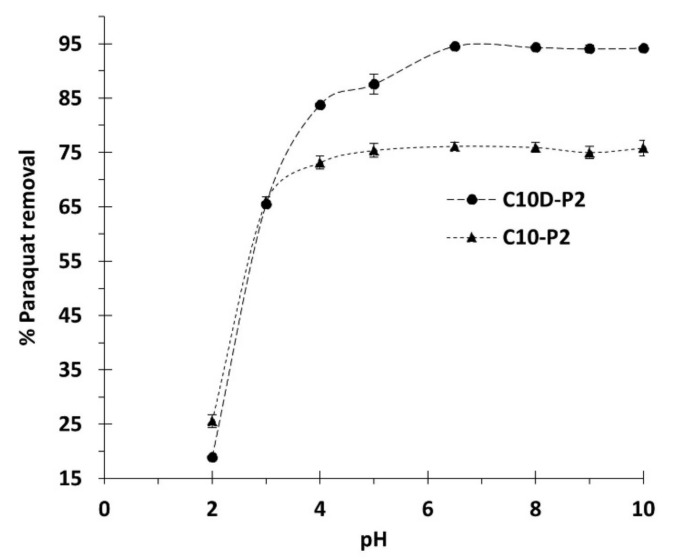
Influence of pH on paraquat removal (conditions: 2 g/L of adsorbent dosage, 25 mg/L of PQ initial concentration, 180 min of contact time, and temperature at 303 K).

As observed in Figure 8, CD10-P2 and CD10-P2 exhibited a low adsorption performance (18.9% and 25.6% of paraquat removal, respectively) at pH 2 because the removal probably occurred due to the inclusion complex, network capture, and bonding. Herein, the electrostatic interaction was restrained because of the deactivation of carboxylic functions into carboxylate groups, where the pH of the solution was inferior to the pKa of citric acid (3.13, 4.76, and 6.40). Consequently, the adsorption was very low, as has also been reported in the literature [48,105,106]. The adsorption efficiency of CD10-P2 was then gradually increased with pH until attaining the maximum at a pH of 6.5 due to the electrostatic interaction [48]. The pH of the solution was superior to the pKa of citric acid, which allowed the total formation of carboxylate functions to interact with the cationic groups of PQ. However, the adsorption of CD10-P2 rose sharply at pH 3 before reaching a plateau at pH 6.5. Therefore, the optimal pH of 6.5 was selected for the next study.

An introductory adsorption experiment was conducted to optimize the effect of a crosslinking agent on the adsorption efficiency of PQ, which is a quaternary ammonium molecule. As remarked in Table 1, the increase of CTR enhanced the paraquat removal up to 94.5% (equal to 11.8 mg/g) for 25 mg/L of initial concentration because of the higher *IEC* value (3.14 mmol/g), which highlighted the presence of an anionic character on nanosponges and confirmed the dominance of ionic interaction between the cationic charge of PQ and the anionic charge of nanosponges. Therefore, the quantity of CTR was selected as 10% *w*/*v* for the next study.

The addition of different quantities of PVOH (0.5, 1 or 2% *w*/*v*) on the nanosponges containing 10% *w*/*v* of CTR affected the adsorption performance. However, the highest PVOH exhibited the lowest *IEC*, but it also demonstrated the highest swelling and β-CD content, which showed outstanding paraquat removal because PQ not only interacted due to the ionic interaction but also due to the other possible processes, as shown in Figure 1. This complementary process was only found by PVOH parts, such as the network capture of PQ in the crosslinked structure (CTR crosslinked PVOH) and hydrogen bonding between the N atoms of PQ and the H atoms of PVOH, which improved the adsorption capacity of PQ. These results were in accordance with the results reported in the literature, which demonstrated that the quantity of aniline extraction was higher with the rise of PVOH embedded in the membrane, even at the low or high initial concentration of aniline [79]. Thus, supplementary removal could distribute an adequate quantity of PVOH on the nanosponges.

#### 3.2.2. Kinetics Study

**Figure 9:**
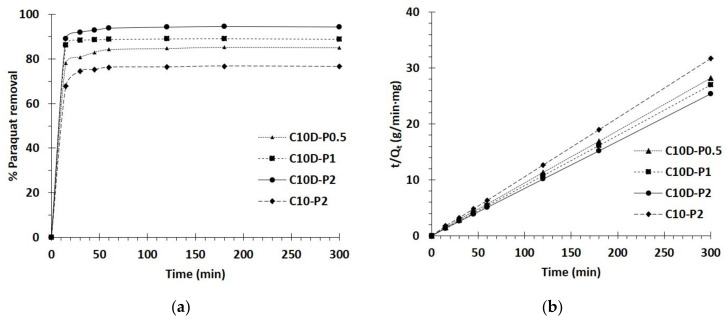
(**a**) Influence of contact time on adsorption capacity; (**b**) Pseudo second-order kinetics of PQ adsorption (conditions: 2 g/L of adsorbent dosage, 25 mg/L of PQ initial concentration, pH of 6.5, and temperature at 303 K).

The kinetics of PQ removal of different nanosponges was performed with various contact times, as seen in Figure 9a. The adsorption increased significantly for the initial 15 min until achieving saturation of adsorption (180 min), according to the lack of vacant active sites. Therefore, a contact time of 180 min was opted for the next study.

The pseudo first-order and second-order models were applied to the experimental data so as to understand the adsorption method relating to a chemical reaction, mass transfer, and adsorption order. As seen in Table 2, the correlation coefficients (R^2^) were superior for the pseudo second-order model (R^2^ = 0.9997, 0.9994, 0.9998, and 0.9991) than the pseudo first-order model (R^2^ = 0.7031, 0.7115, 0.7133, and 0.7541) for these nanosponges, respectively: C10D-P0.5, C10D-P1, C10D-P2, and C10-P2. The pseudo second-order model showed a straight line, in which the correlation coefficient was closed to 1, and confirmed the suitability of the model with experimental data, as illustrated in Figure 9b. Therefore, the adsorption capacity at equilibrium, estimated by the pseudo second-order model (Q_e,cal_ = 10.7, 11.1, 11.8 and 9.5 mg/g) was virtually identical to the experimental value (Q_e,exp_ = 10.6, 11.1, 11.8, and 9.6 mg/g) for the nanosponges C10D-P0.5, C10D-P1, C10D-P2, and C10-P2, respectively.

The diffusion mechanism during adsorption was studied by the intra-particle diffusion model, which was divided into two parts. The adsorption rate constant was higher for the first step (k_31_) than for the second step (k_32_) for every nanosponge, as observed in Table 2. The fast adsorption was mainly attributed to the boundary layer diffusion and the slow adsorption was due to the intraparticle diffusion. The curve of the two sections did not pass through the origin, which proved that paraquat adsorption was a complicated procedure, as published in the literature [107,108].

#### 3.2.3. Isotherm Study

**Figure 10:**
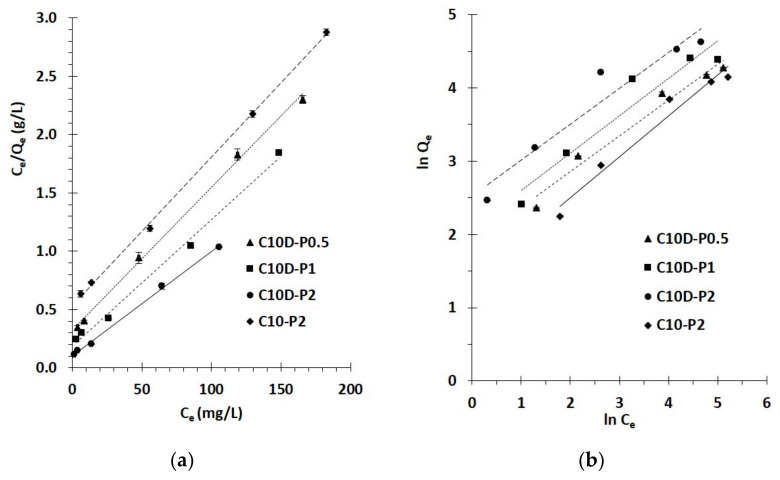
(**a**) Langmuir isotherm; (**b**) Freundlich isotherm of PQ adsorption (conditions: 2 g/L of adsorbent dosage, 180 min of contact time, pH of 6.5, and temperature at 303 K).

The interaction between PQ and nanosponges at equilibrium state was investigated by an isotherm study with different initial concentrations of PQ (from 25 to 300 mg L^−1^) at 30 °C. The Freundlich and Langmuir isotherm models were applied to the experimental data, as shown in Figure 10a,b, respectively. The estimation of isotherm parameters is shown in Table 3. The correlation coefficient (R^2^) was much higher for the Langmuir isotherm model (R^2^ = 0.9975, 0.9939, 0.9979, and 0.9990) than the Freundlich isotherm model (R^2^ = 0.9706, 0.9245, 0.9307, and 0.9665) for the nanosponges C10D-P0.5, C10D-P1, C10D-P2, and C10-P2, respectively. The correlation coefficient value of the Langmuir isotherm model near to 1 indicated a linear relationship, which asserted the reasonableness of the model with experiment data. This isotherm also described the monolayer adsorption for PQ, which dominated on the regular surface of nanosponges. The chi-square values for the Langmuir model (1.2, 1.6, 1.0, and 3.0) were inferior to the Freundlich isotherm model (6.9, 1.9, 2.8, and 12.9) for C10D-P0.5, C10D-P1, C10D-P2, and C10-P2, respectively, which suggests that the experimental data was suitable with the Langmuir isotherm. Ultimately, the separation factor (R_L_) was decreased with the increase of initial concentrations, which revealed a strong affinity between PQ and nanosponges if 0 < R_L_< 1.

In Table 4, the cyclodextrin nanosponges could be applied as an efficient adsorbent for paraquat removal from water, which displayed the maximum adsorption capacity of 112.2 mg/g. However, it was listed at the fourth-best adsorption capacity after calixarene, magnetic adsorbent, and carbon nanotubes.

#### 3.2.4. Reusability Study

**Figure 11:**
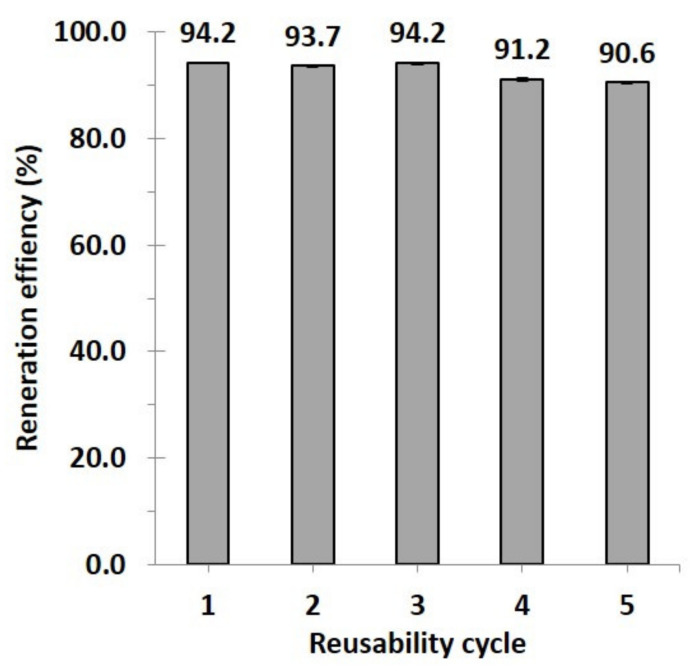
Reusability of C10D-P2 nanosponges (conditions: 2 g/L of adsorbent dosage, pH of 6.5, and temperature at 303 K).

For economic and effective reasons in the adsorption process, the reusability of C10D-P2 nanosponges was performed in methanol to release PQ and renew the nanosponges. The regeneration efficiency achieved 90.3% after five cycles, as observed in Figure 11.

## 4. Conclusions

Anionic nanosponges were obtained by crosslinking citric acid (10% *w*/*v*) and β-cyclodextrin (10% *w*/*v*) in the presence of poly(vinyl alcohol) (2% *w*/*v*), which resulted in 60.2% yield, 3.14 mmol/g ion exchange capacity, 0.335 mmol/g β-CD content, and 94.5% paraquat removal for 25 mg/L of PQ initial concentration. The presence of new ester bonds on the nanosponges was proven by different spectroscopic techniques. The adsorption of PQ onto the nanosponges occurred due to four possibilities: (i) inclusion complex of PQ into the β-CD cavity, (ii) network capture of PQ on the reticulated framework, (iii) electrostatic interaction of PQ with anionic charge, or (iv) hydrogen bonding of PQ with OH groups. The rise of CTR enhanced the ionic exchange capacity, which increased the adsorption capacity towards PQ. Although the addition of PVOH reduced the anionic character, it also enhanced the PQ adsorption by hydrogen bonding using the long chain of PVOH and the crosslinked structure of PVOH. Thus, the appearance of PVOH was significant to create a novel type of cyclodextrin nanosponge. Adsorption kinetics were achieved at 180 min with the pseudo second-order model. The adsorption isotherm was suitable for the Langmuir model (Q = 112.2 mg/g). Finally, the regeneration performance of nanosponges in methanol was 90.3% after five regeneration times. These environmentally friendly nanosponges could be applied as a valuable green adsorbent to eliminate cationic substances polluted in water.

## Figures and Tables

**Table 1:** Physicochemical properties of poly(vinyl alcohol)-cyclodextrin nanosponges and their derivatives.

Nanosponges	Composition (% *w*/*v*)			% Yield		*IEC* (mmol/g)		% Swelling		% Paraquat Removal		β-CD Content (mmol/g)	
	β-CD	CTR	PVOH	Mean	S.D.	Mean	S.D.	Mean	S.D.	Mean	S.D.	Mean	S.D.
C2.5D-P2	10	2.5	2	52.9	2.0	2.13	0.02	199.2	1.2	66.0	1.9	0.365	0.003
C5D-P2	10	5	2	57.5	1.1	2.93	0.01	147.2	1.4	84.2	0.5	0.351	0.005
C10D-P2	10	10	2	60.2	2.1	3.14	0.03	96.4	0.5	94.5	0.1	0.335	0.003
C10D-P1	10	10	1	63.4	1.5	3.20	0.02	101.6	0.7	89.0	0.2	0.328	0.002
C10D-P0.5	10	10	0.5	68.6	1.3	3.27	0.03	104.1	0.8	85.2	0.5	0.314	0.004
C10-P2	0	10	2	39.9	2.6	3.84	0.05	135.3	0.6	75.9	0.8	-	-
C10-P1	0	10	1	21.3	2.3	4.07	0.03	143.6	0.4	69.2	1.0	-	-
C10-P0.5	0	10	0.5	17.3	1.3	4.46	0.04	166.7	0.9	21.4	0.4	-	-

**Table 2:** Pseudo second-order and pseudo first-order kinetics parameters (conditions: 2 g/L of adsorbent dosage, 25 mg/L of PQ initial concentration, pH of 6.5, and temperature at 303 K).

Nanosponges	Q_e (exp)_	Pseudo First-Order			Pseudo Second-Order					Adsorption Mechanism	
		R^2^	Q_e (cal)_	k_1_	R^2^	Q_e (cal)_	k_2_	h	t_1/2_	k_31_	k_32_
C10D-P0.5	10.6	0.7031	2.8	0.0104	0.9997	10.7	0.1248	14.2	0.8	0.2011	0.0163
C10D-P1	11.1	0.7155	3.0	0.0111	0.9994	11.1	0.1670	20.7	0.5	0.1511	0.0148
C10D-P2	11.8	0.7133	3.1	0.0117	0.9998	11.8	0.4709	65.8	0.2	0.0807	0.0131
C10-P2	9.6	0.7541	2.1	0.0064	0.9991	9.5	0.1126	10.2	0.9	0.2640	0.0217

**Table 3:** Langmuir and Freundlich isotherm parameters (conditions: 2 g/L of adsorbent dosage, 180 min of contact time, pH of 6.5, and temperature at 303 K).

Nano-Sponges	Q_m (exp)_	Langmuir Isotherm									Freundlich Isotherm				
		R^2^	Q_m (cal)_	K_L_	χ^2^	R_L_ for C_0_ (mg/L)					R^2^	Q_m (cal)_	K_f_	1/n	χ^2^
						25	50	150	250	300					
C10D-P0.5	71.9	0.9975	82.0	0.04	1.2	0.51	0.34	0.15	0.10	0.08	0.9706	52.9	6.5	0.49	6.9
C10D-P1	80.5	0.9939	92.6	0.06	1.6	0.41	0.26	0.10	0.07	0.05	0.9245	69.2	7.4	0.51	1.9
C10D-P2	102.0	0.9979	112.4	0.09	1.0	0.32	0.19	0.07	0.04	0.04	0.9307	120.4	12.5	0.49	2.8
C10-P2	63.5	0.9990	78.7	0.02	3.0	0.63	0.46	0.22	0.14	0.12	0.9665	40.6	4.0	0.56	12.9

**Table 4:** Langmuir isotherm for paraquat removal by various adsorbents.

Adsorbent	Adsorption Dosage	Paraquat Concentration (mg/L)	Maximum Adsorption Capacity
PVOH-Cyclodextrin nanosponges (This work)	0.02 g in 0.01 L	25–250 mg/L	112.2 mg/g
Cyclodextrin nanosponges [48]	0.02 g in 0.01 L	10–200 mg/L	21.9 mg/g
Cellulose nanofiber [46]	0.02 g in 0.2 L	-	108 g/g
Montmorillonite [45]	0.002 g in 0.1 L	-	0.44 mol/kg
Magnetic adsorbent [42]	0.0025 g in 0.005 L	30–900 mg/L	242.2 mg/g
Carbon nanotubes [38]	0.002 g in 0.005 L	70–250 mg/L	218.6 mg/g
Graphene oxide [37]	0.02 g in 0.025 L	4–24 mg/L	29.15 mg/g
Calixarene [34]	0.025 g in 0.005 L	0.5–2.0 mmol/L	419 mg/g
Activated carbon [29]	0.01 g in 0.01 L	1.5–45 mg/L	20 mg/g
Microorganisms [28]	0.005 g in 0.015 L	0–285.7 mg/L	24.4 mg/g
Bentonite [27]	0.04 g in 0.025 L	4–24 mg/L	11.75 mg/g
Bio-based material [23]	0.069 g in 0.025 L	25–85 mg/L	20.58 mg/g

## Data Availability

The study did not report any data.
